# Duration of Exposure to Elevated Temperature Affects Competitive Interactions in Juvenile Reef Fishes

**DOI:** 10.1371/journal.pone.0164505

**Published:** 2016-10-13

**Authors:** Donald T. Warren, Jennifer M. Donelson, Mark I. McCormick, Maud C. O. Ferrari, Philip L. Munday

**Affiliations:** 1 Department of Marine Biology and Aquaculture, James Cook University, Townsville, Queensland, 4811 Australia; 2 ARC Centre of Excellence for Coral Reef Studies, James Cook University, Townsville, Queensland, 4811 Australia; 3 School of Life Sciences, University of Technology Sydney, PO Box 123, Broadway, New South Wales, 2007 Australia; 4 Department of Biomedical Sciences, WCVM, University of Saskatchewan, Saskatoon, Canada; Leibniz Center for Tropical Marine Ecology, GERMANY

## Abstract

Climate change will affect key ecological processes that structure natural communities, but the outcome of interactions between individuals and species will depend on their thermal plasticity. We tested how short- and long-term exposure to projected future temperatures affects intraspecific and interspecific competitive interactions in two species of coral reef damselfishes. In conspecific contests, juvenile Ambon damselfish, *Pomacentrus amboinensis*, exhibited no change in aggressive interactions after 4d exposure to higher temperatures. However, after 90d of exposure, fish showed a nonadaptive reduction in aggression at elevated temperatures. Conversely, 4d exposure to higher temperature increased aggression towards conspecifics in the lemon damselfish, *Pomacentrus moluccensis*. 90d exposure began to reduce this pattern, but overall there was little effect of temperature. Aggression in interspecific contests increased with short-term exposure, but was significantly lower after long-term exposure indicative of acclimation. Our results show how the length of exposure to elevated temperature can affect the outcome of competitive interactions. Furthermore, we illustrate that results from intraspecific contests may not accurately predict interspecific interactions, which will challenge our ability to generalise the effects of warming on competitive interactions.

## Introduction

Climate change will alter the physiology, behaviour, and geographical distribution of many species [1–3]. However, the impact of these changes on population and community structure will depend on the outcome of biological interactions with other species [4,5]. Due to differences in thermal tolerances, some species will be more capable of maintaining performance at higher temperatures than others [6,7]. Consequently, differences in thermal tolerance could dramatically alter the outcome of ecological interactions, such as competition and predation [8,9]. Yet, thermal tolerances can change if exposure to new thermal conditions occurs for a sufficient amount of time at critical periods [10,11]. Furthermore, the extent of phenotypic change can depend on the length of exposure, with longer-term periods believed to unlock greater plasticity [12,13]. While many studies are now investigating the effects of higher temperatures on physiological processes [14–16], incorporating longer exposure lengths during early life has received much less attention. In climate change studies, relevant exposure will be critical for predicting the outcome of ecological processes in a future warmer world [3,13,17].

Much of our understanding of how species might respond to future warming comprises of relatively short-term experiments using adults [18,19]. Yet, exposure of juveniles to higher temperatures during early development can potentially induce greater plasticity in phenotypic traits and greater thermal tolerance in later life stages than just exposure of adults [20–23]. These early life influences can then produce long-lasting alterations in phenotype, affecting individual success later in life [24]. Changes in phenotype that prove beneficial to an individual are known as acclimation and could be a method to maintain performance in a new environment [25]. However, acclimation may not always fully compensate for the negative effects of environmental stress and can result in partial acclimation or even overcompensation [26,27]. Furthermore, not all induced changes will be beneficial to future performance [28,29]. Instead, prolonged exposure to elevated temperature may result in a further degrade in performance compared to present-day levels [30–32]. Understanding the influence of early environmental experience is important, because early life stages are critical in population regulation [33]. Many juveniles are vulnerable to resource restriction and even small changes in resource acquisition can affect attributes like growth and survival [34,35]. At this age, competitive interactions greatly influence which individuals will survive to the next life stage [36,37].

Performance in tropical ectotherms is believed to be particularly sensitive to higher temperature as they occupy relatively stable thermal environments and often live close to their thermal maximums [38,39]. Small increases in temperature may have large effects on competitive performance in these environments [40]. However, the effect of longer-term exposure to elevated temperatures on competitive ability has not been tested. The objective of the present study was to determine how the length of exposure to elevated thermal conditions in post-settlement recruits affects the outcome of competitive interactions within and between two species of coral reef damselfish, *Pomacentrus moluccensis* and *P*. *amboinensis*. These species are known competitors for shelter over a broad geographic range [41], making them ideal candidates for this study. We raised juveniles of both species at three temperatures and two exposure lengths to test: (1) the effects of elevated temperature on aggressive interactions and (2) compare performance from short-term to long-term exposure to higher temperature. We expected that short-term exposure would have a negative impact on competitive interactions by reducing aggression in contests. After long-term exposure, we predicted either acclimation to occur and aggression would be restored towards control levels, or that extended exposure would accumulate stress and lead to a further decline in aggression performance.

## Methods

### Study species, collection, and holding facilities

The study species were the lemon damselfish, *P*. *moluccensis*, and the Ambon damselfish, *P*. *amboinensis*. These species are commonly used in a wide range of ecological, behavioural and physiological experiments [41–43]. They co-occur in the same habitat for the majority of their geographic range, spanning from the Coral Sea to Southeast Asia [44]. Much research undertaken on their interactions has been done at Lizard Island on the northern Great Barrier Reef, Australia (-14°67´S, 145°44´E). Both species prefer to settle to live coral [45,46] and in the wild *P*. *moluccensis* is exclusively found on live coral, while *P*. *amboinensis* is found on a broader range of habitats including dead coral and rubble [41,46]. These species feed on similar food items as juveniles [41] and *P*. *moluccensis* is normally outcompeted for preferred habitat and is forced to occupy a position near the top of a coral head, while *P*. *amboinensis* occupies the safer bottom part of the habitat patch [47]. Body size is strongly related to dominance within [46,48] and between the two species [41]. All work reported herein was conducted under permits from James Cook University Animal Ethics Committee (A2079), the Great Barrier Reef Marine Park Authority (G10/33239.1), and Queensland Fisheries (170251).

Like many reef fishes, these species have a pelagic larval phase that prevents rearing individuals from their embryonic phase. After 3–4 weeks, larvae make their final metamorphosis and recruit back as juveniles to join the reef community. This transition period is an important life history bottleneck as there are more juveniles than available shelter, forcing individuals to compete for space [49]. Collection efforts targeted the smallest (~20 mm standard length), and therefore youngest of these recruits. Fish were collected from reefs in the Cairns region (-16°78´S, 146°26´E) of the Great Barrier Reef, Australia during January 2014 and transferred to experimental facilities at James Cook University. Individuals were randomly allocated to replicate 40 litre tanks in three temperature treatments: 29°C (current-day summer average for the collection region; control; [50]), 30°C, and 31°C (projected future temperatures by 2100; [51]). Elevated temperature treatments were split into two exposure lengths: 4d or 90d. We used 4d for our short-term exposure to explore the impacts of elevated temperature on competitive behaviour without causing a thermal stress response [52]. Previous work using *P*. *moluccensis* has shown no acclimation to similar elevated temperatures to occur for up to 22 days, supporting that our 4d treatment would not be confounded by reversible acclimation [53]. We chose a 90d exposure for our long-term treatment based on previous developmental studies using this species [54] and another closely related species of damselfish [55]. Daily temperature variation and photoperiods followed a natural cycle for the collection region, ± 0.6°C around the mean and 12:12h, respectively [50].

### Experimental design

To determine the effects of short-term exposure to elevated temperature on competitive interactions we conducted intraspecific and interspecific trials with fish from 29°C tested at 30°C, and 31°C. Holding tank temperatures were raised 1°C/day (to reduce the effect of heat shock; [52,53]) and held at target temperature for 4d before testing. To determine the effects of long-term thermal exposure on competitive interactions, we conducted intraspecific and interspecific trials at 30°C and 31°C using fish that had been maintained at these temperatures for 90d.

Our control treatments maintained fish at 29°C and tested at 29°C. All competitive trials were conducted during one experimental period to ensure fish were the same age. No trials occurred between fish from the same holding tank to control for the possibility of a pre-established hierarchy and no two competitors were matched more than once to prevent a winner effect. In total there were 40 holding tanks for the five treatment combinations: control, two combinations for short-term exposure (30 and 31°C), and two combinations for long-term exposure (30 and 31°C; S1 Table). Ten replicates were conducted for each treatment combination.

### Competitive interaction trials

**Fig 1:**
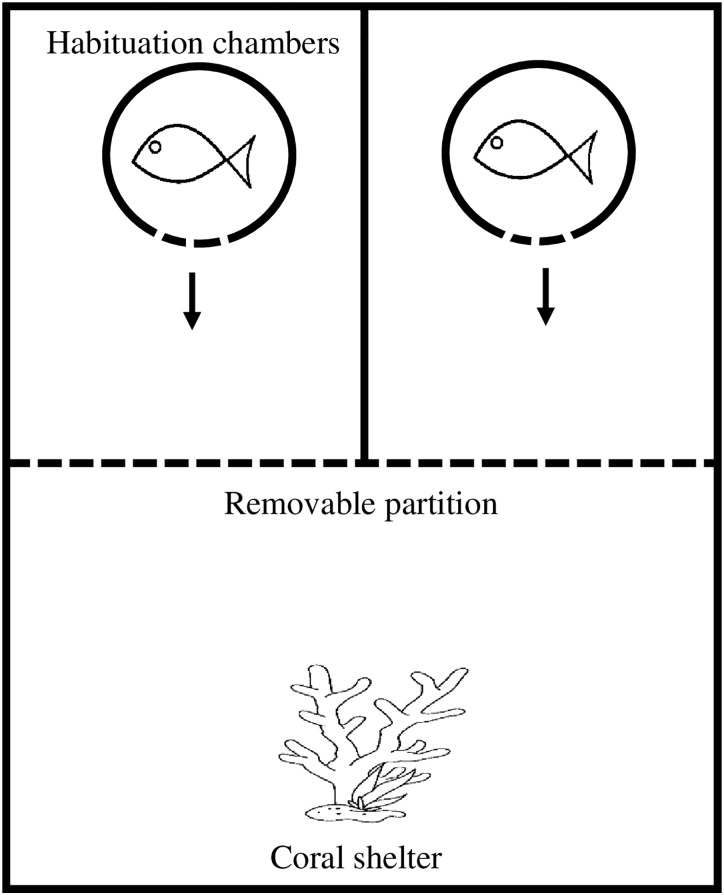
Experimental setup for competition trials. Fish began in habituation chambers (circles) for 10 min. Revolving doors were opened and fish were allowed to emerge. Once emerged, a second partition was raised (dotted line) exposing fish to each other and a coral skeleton for shelter. A 10 min video recording was taken of their interactions.

Size difference between competitors is a known factor in determining the outcome of competitive contests [41]. Consequently, all fish were measured just before the experimental period (standard length mm, $\bar{x}=\;29.1\;\pm \;4.1\text{ SD}$) and pairs were created by matching fish within 10% of their standard length [56]. Competitive arenas and procedures followed Killen *et al*. [57]. At the start of the trial, fish were placed individually in habituation chambers for 10 min (Fig 1), consisting of PVC cylinders with a revolving door. After a 10 min habituation period, the doors on both chambers were carefully opened simultaneously and fish were allowed to emerge. Once both fish exited their habituation chambers, a central partition was raised, exposing both fish to one another as well as to a fragment of coral skeleton (~5 x 5 x 5 cm). The coral provided shelter and also served as a resource for competition. Competitive interactions were video recorded for 10 min and later analysed for three behavioural traits: (i) displays, defined as a lateral flare of its fin towards the opponent; (ii) attacks, defined as a chase or biting of the opponent; and (iii) avoidances, swimming away from an opposing attack or display. These variables were used to calculate an aggression score = attacks + displays−avoids [58]. The fish with the higher aggression score was deemed dominant and the winner for that pair. Aggression was used as a measure for competitive performance as it provides a good indicator of contest outcomes [57].

### Data analysis

Size differences for each pair, calculated as the size of the winner fish minus the size of the loser fish, were normally distributed and did not differ from zero (one-sample t-test, *t*_150_ = 0.213, *p* = 0.832). This indicated size matching was successful and the remaining size differences had no effect on the outcome of contests. Subsequent analyses were performed without correcting for any size differences. For each trial, individual aggression scores were calculated and the difference of the winner minus the loser score was computed. Aggression score differences were analysed with separate ANOVAs to measure the effect of short-term exposure to elevated temperature and to compare short-term with long-term exposure. This was repeated for each species in the intraspecific treatments and the interspecific treatment for a total of six models. Preliminary analysis included holding tank in the model to test for tank effect, but no effect was found. Consequently, results reported do not include this variable in the design.

While increased temperature could affect the difference in aggression score between competitors, it could also affect the absolute level of aggression as well as the total number of aggressive interactions during a trial. To ensure aggression score difference was an accurate reflection of overall aggressive behaviour, aggression score of winner fish only and total number of aggressive interactions were analysed similar to aggression score differences. For interspecific trials, proportion of wins by species were compared across temperature and exposure length with a chi-square test of independence.

## Results

### Intraspecific competition

**Fig 2:**
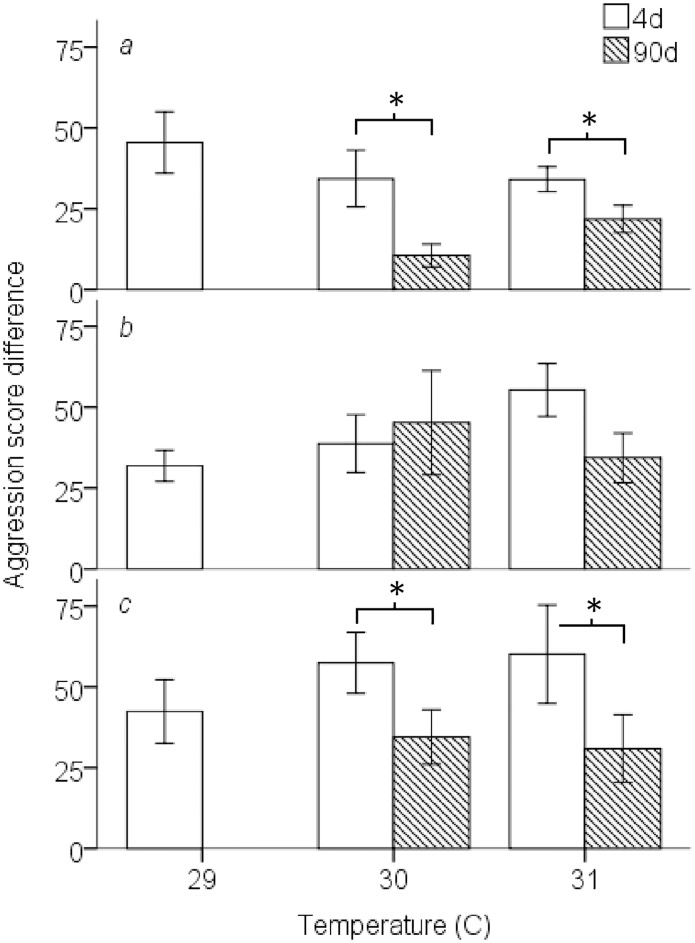
Mean aggression score difference ± SE, calculated as winner fish score minus loser score, between competitors by temperature of 4d (grey) and 90d exposure treatments (open). Species composition listed by row: intraspecific *Pomacentrus amboinensis* (*a*), intraspecific *P*. *moluccensis* (*b*), and interspecific (*c*). Statistical significance (*p* < 0.05) between 4d and 90d treatments represented with (*). All treatments n = 10.

For *P*. *amboinensis*, there was no change in aggression score difference following 4d exposure to higher temperatures (Fig 2a). After 90d, fish had a significantly smaller difference in aggression scores at 30°C and 31°C compared to the 4d treatment (F_1,38_ = 10.7, P = 0.002; Fig 2a). For *P*. *moluccensis*, there was a trend to increase aggression score differences following 4d exposure (Fig 2b) and if we only considered the aggression score of the winner, this effect was significant (F_2,27_ = 3.46, P = 0.046; S1 Fig). There was no difference in aggression score differences between 4d and 90d (Fig 2b). The aggression score of the winner only (S1 Fig), and the total number of aggressive interactions by the two individuals (S2 Fig), mirrored aggression score differences in both the 4d and 90d treatments confirming aggression score differences were an appropriate measure of aggressive behaviour during a trial.

### Interspecific competition

There was no change in aggression score differences among temperature treatments for contests with 4d exposure, although there was a tendency for the aggression scores to increase with temperature (Fig 2c). Fish with 90d of exposure had lower aggression score differences at elevated temperatures compared with 4d (F_1,38_ = 5.14, P = 0.029; Fig 2c). The aggression score of the winner only (S1 Fig), and total number of aggressive interactions by the two individuals (S2 Fig), mirrored aggression score differences in both the 4d and 90d treatments.

**Fig 3:**
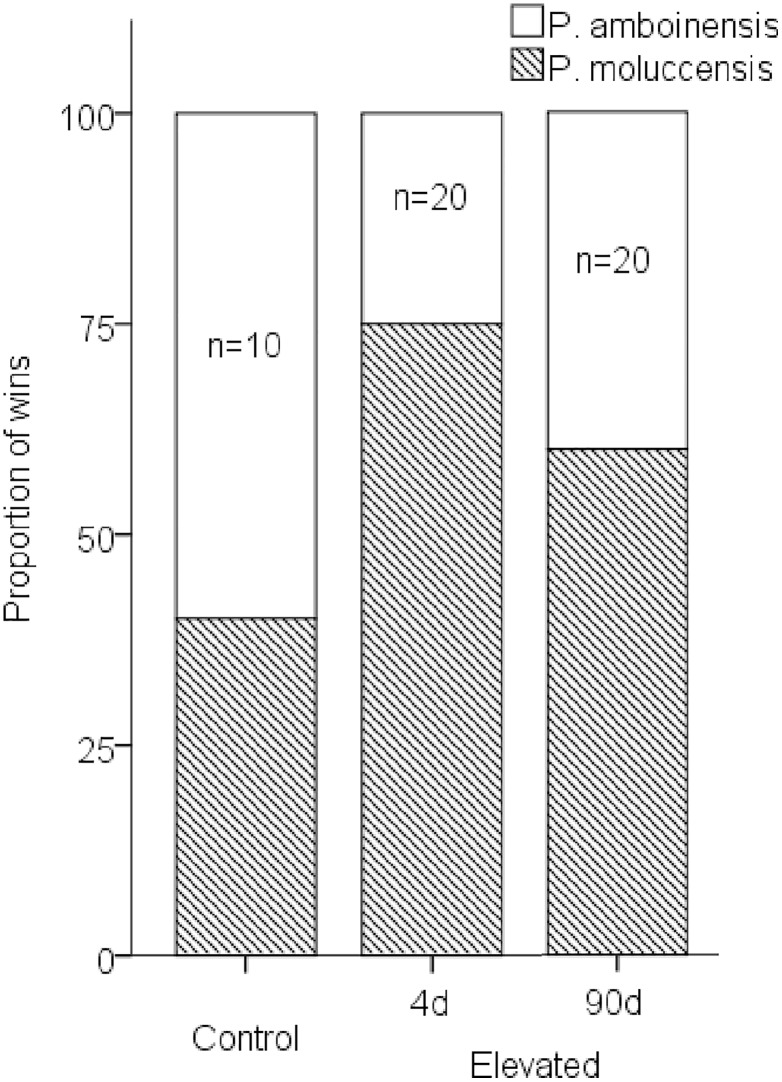
Proportion of wins by species for interspecific contests. *Pomacentrus moluccensis* (grey) and *P*. *amboinensis* (open) tested in “control” (29°C) and “elevated” (30 + 31°C combined) temperature treatment. Elevated temperature treatments are split by 4d and 90d exposure.

There was no significant difference in the proportion of contests won by species across the three temperatures after 4d exposure. However, when the 30 and 31°C treatment groups were combined into an “elevated temperature” group, there was a trend from favouring *P*. *amboinensis* at control temperatures to favouring *P*. *moluccensis* in elevated temperatures (*χ*^2^ = 3.5, *df* = 1, *p* = 0.07; Fig 3). After 90d exposure, this trend was reduced, though proportions did not fully return to controls.

## Discussion

Climate change is predicted to affect the outcome of ecological interactions within and between species, with potentially far-reaching consequences for population dynamics and community structure [59]. Elevated temperatures can lead to negative effects on individual performance [1], but individual performance may acclimate and return to control levels after long-term exposure to these temperatures [12,25]. Alternatively, longer exposure could result in a further decline in performance [32,29]. We found aggression after short-term exposure was maintained in intraspecific contests with *P*. *amboinensis*, whereas there was a trend towards increasing aggression in *P*. *moluccensis*. Long-term exposure reduced aggression scores in *P*. *amboinensis*, but *P*. *moluccensis* was unchanged. Interestingly, the aggressive response of species in interspecific contests differed from what would have been predicted from each species’ performance in intraspecific contests. This suggests that predicting the outcomes of species interactions in the future may not be possible from measuring the behavioural performance of species independently.

Elevated temperatures influenced the outcome of competitive interactions for interspecific contests. There was a tendency for increased aggression score differences with short-term exposure to elevated temperature. After long-term exposure, acclimation appears to have occurred as aggression was significantly lower compared to fish with short-term exposure. In fact, acclimation may have overcompensated as aggression was lower than control in the long-term treatment. Exposure length also appeared to influence the relative proportions of wins by each species. While *P*. *amboinensis* was favoured in control conditions, *P*. *moluccensis* tended to win more contests after short-term exposure to elevated temperatures. This trend was less apparent with long-term exposure, though proportions did not fully return to controls. Changes in species dominance at elevated temperature can occur in competitive hierarchies of both marine [60] and freshwater species [61], but whether these changes are diminished or reversed with longer-term exposure during early life has not been tested.

Elevated temperature had different effects on each species for intraspecific competition, possibly due to differential thermal sensitivity and performance optima. Temperature had only small effects on competitive interactions in *P*. *moluccensis*. Short-term exposure had an increasing trend with temperature, while long-term exposure showed a relatively flat response with the biggest difference between exposure lengths at 31°C. The lowered aggression with longer exposure at 31°C matches previous research showing evidence of acclimation in aerobic capacity for this species, from a nearby reef region, after early development in similar temperatures [54]. These results suggest this species may fare well within the +2°C future temperature increases. In contrast, *P*. *amboinensis* maintained aggression levels with short-term exposure, but long-term exposure resulted in a reduction of aggressive performance. This indicates there was no acclimation to elevated temperature. Instead, this shows how prolonged exposure will not always induce phenotypic changes that are beneficial to an individual’s performance. Other studies have also reported non-beneficial, or non-adaptive, responses after long-term exposure periods to temperature [26,31,62]. These non-adaptive responses have been attributed to insufficient duration of exposure for acclimation to occur [63], conditions too extreme for any acclimation to occur [29], or costs of acclimation outweighing its benefits [64]. Alternatively, the longer exposure period could have resulted in accumulated physiological stress that caused performance to decline. In our study, one or more of these may have acted to create a non-beneficial response.

As global temperatures continue to rise, individuals will be exposed to increased temperatures throughout their early life stages. Thermal plasticity will likely be a key process in determining performance in ecological interactions in future warmer environments. This is the first study to show that extended exposure to elevated temperature affects competitive interactions in coral reef fishes. Our intraspecific results suggest that *P*. *amboinensis* should perform poorly relative to *P*. *moluccensis* in interspecific contests at elevated temperature. While this was true for short-term treatments, we found this trend was less apparent after long-term exposure. This highlights that interspecific competitive interactions can be complex and extrapolation of results from within species to predicting between species contests may not be accurate. Future studies should also consider exposure periods similar to, if not longer than, our study and be combined with physiological measurements on stress responses when attempting to project the outcome of competitive interactions between species in a future warmer world.

## Supporting Information

S1 Dataset Dataset listing counts of behaviours (Attacks, Displays, and Avoids) during competitive interaction trials. Behaviours were used to calculate an “Aggression score” = Attacks + Displays−Avoids. Rows are grouped in pairs, one for each competitor. “Diff, Total interactions, and Aggression difference” reference the competitor from the same row and the row beneath as a pair. Diff = the difference in “Size” for the pair. Total interactions = sum of the “Attack”, “Display”, and “Avoid” for the pair. Aggression difference = absolute value of the difference in “Aggression scores” for the pair.
(XLSX)

S1 Fig Aggression scores ± SE of contest winner. Test temperature listed horizontally and split by 4d (grey) and 90d exposure treatments (open). Intraspecific contests of *Pomacentrus amboinensis* (*a*) with after 4d exposure to elevated temperature had no change in winner aggression score compared to controls, but 90d exposure had significantly lower winner scores when compared to 4d (F_1,38_ = 7.79, P = 0.008). Aggression scores for *Pomacentrus moluccensis* (*b*) increased from control after 4d exposure to elevated temperature (F_2,27_ = 3.46, P = 0.046), but there was no difference between 4d and 90d. Aggression in interspecific contests (*c*) increased slightly with temperature after 4d, but was reduced to control levels after 90d exposure (F_1,38_ = 5.14, P = 0.029). Significance of p < 0.05 symbolised with (*).
(DOCX)

S2 Fig Total number of interactions ± SE by both competitors per contest. Calculated as sum of attacks, displays, and avoids for both competitors. Test temperature listed horizontally, and split by 4d (grey) and 90d exposure treatments (open). Intraspecific contests of *Pomacentrus amboinensis* (*a*) had a non-significant reduction of interactions after 4d exposure to temperature compared to controls but 90d exposed treatments had significantly less interactions than 4d (F_1,38_ = 4.17, P = 0.048). Contests with *Pomacentrus moluccensis* (*b*) had more interactions with elevated temperature after 4d, but showed no difference after 90d. Interspecific treatments (*c*) had increased interactions with temperature after 4d, but 90d exposure reduced this back to control levels (F_1,38_ = 6.3, P = 0.016). Significance P < 0.05 symbolised with (*).
(DOCX)

S1 Table Experimental design matrix. Aim 1 compared control (grey) fish to individuals with 4d exposure (solid) to elevated temperatures. Aim 2 compared contests using 4d exposed fish with 90d exposure treatments (open).
(DOCX)

S2 Table Statistical summary of one-way ANOVA analyses. Separate tests were generated for the effects of short-term exposure to elevated temperature (left) and comparing exposure durations at elevated temperature (right) on (a) aggression score differences, (b) aggression score of contest winner, and (c) total number of interactions for 3 species combinations.
(XLSX)

## References

[1] Parmesan C. Ecological and evolutionary responses to recent climate change. Annual Review of Ecology, Evolution, and Systematics. 2006; 37: 637–669.

[2] Botkin DB, Saxe H, Araujo MB, Betts R, Bradshaw RH, Cedhagen T. Forecasting the effects of global warming on biodiversity. Bioscience. 2007; 57: 227–236. 10.1641/B570306

[3] Huey RB, Kearney MR, Krockenberger A, Holtum JA, Jess M, Williams SE. Predicting organismal vulnerability to climate warming: roles of behaviour, physiology and adaptation. Philosophical Transactions of the Royal Society of London B: Biological Sciences. 2012; 367: 1665–1679. 10.1098/rstb.2012.0005 22566674 PMC3350654

[4] Hughes L. Biological consequences of global warming: is the signal already apparent? Trends in Ecology & Evolution. 2000; 15: 56–61. 10.1016/S0169-5347(99)01764-4 10652556

[5] Harley CD, Randall Hughes A, Hultgren KM, Miner BG, Sorte CJ, Thornber CS et al The impacts of climate change in coastal marine systems. Ecology Letters. 2006; 9: 228–241. 10.1111/j.1461-0248.2005.00871.x 16958887

[6] Milazzo M, Mirto S, Domenici P, Gristina M. Climate change exacerbates interspecific interactions in sympatric coastal fishes. Journal of Animal Ecology. 2012; 8: 468–477. 10.1111/j.1365-2656.2012.02034.x 23039273

[7] Pörtner H. Climate change and temperature-dependent biogeography: oxygen limitation of thermal tolerance in animals. Naturwissenschaften 2001; 88: 137–146. 10.1007/s001140100216 11480701

[8] Walther GR, Post E, Convey P, Menzel A, Parmesan C, Beebee TJ et al Ecological responses to recent climate change. Nature. 2002; 416: 389–395. 10.1038/416389a 11919621

[9] Pörtner HO, Farrell AP. Physiology and climate change. Science. 2008; 322: 690–692. 10.1126/science.1163156 18974339

[10] Huey RB, Kinsolver JG. Evolution of thermal sensitivity of ectotherm performance. Trends in Ecology & Evolution. 1989; 4: 131–135. 10.1016/0169-5347(89)90211-5 21227334

[11] Schulte PM, Healy TM, Fangue NA. Thermal performance curves, phenotypic plasticity, and the time scales of temperature exposure. Integrative and Comparative Biology. 2011; 1–13. 10.1093/icb/icr097 21841184

[12] Angilletta MJ. Thermal adaptation: a theoretical and empirical synthesis. Oxford University Press; 2009

[13] Munday PL, Warner RR, Monro K, Pandolfi JM, Marshall DJ. Predicting evolutionary responses to climate change in the sea. Ecology Letters. 2013; 16: 1488–1500. 10.1111/ele.12185 24119205

[14] Nilsson GE, Crawley N, Lunde IG, Munday PL. Elevated temperature reduces the respiratory scope of coral reef fishes. Global Change Biology. 2009; 15: 1405–1412. 10.1111/j.1365-2486.2008.01767.x

[15] Johansen JL, Jones GP. Increasing ocean temperature reduces the metabolic performance and swimming ability of coral reef damselfishes. Global Change Biology. 2011; 17: 2971–2979. 10.1111/j.1365-2486.2011.02436.x

[16] Mora C, Ospina AF. Tolerance to high temperatures and potential impact of sea warming on reef fishes to Gorgona Island (tropical eastern Pacific). Marine Biology. 2001; 139: 765–769. 10.1007/s002270100626

[17] Sentis A, Morisson J, Boukal DS. Thermal acclimation modulates the impacts of temperature and enrichment on trophic interaction strengths and population dynamics. Global Change Biology. 2015; 21: 3290–3298. 10.1111/gcb.12931 25808556

[18] Seebacher F, Holmes S, Roosen NJ, Noucian M, Wilson RS, Ward AJW. Capacity for thermal acclimation differs between populations and phylogenetic lineages within a species. Functional Ecology. 2012; 26: 1418–1428. 10.1111/j.1365-2435.2012.02052.x

[19] Johnson T, Bennett A. The thermal acclimation of burst escape performance in fish: an integrated study of molecular and cellular physiology and organismal performance. The Journal of Experimental Biology. 1995; 198: 2165–2175. 9320080 10.1242/jeb.198.10.2165

[20] Scott GR, Johnston IA. Temperature during embryonic development has persistent effects on thermal acclimation capacity in zebrafish. Proceedings of the National Academy of Sciences. 2012; 109: 14247–14252. 10.1073/pnas.1205012109 22891320 PMC3435178

[21] Donelson JM, Munday PL, McCormick MI, Nilsson GE. Acclimation to predicted ocean warming through developmental plasticity in a tropical reef fish. Global Change Biology. 2011; 17: 1712–1719. 10.1111/j.1365-2486.2010.02339.x

[22] Tracy RL, Walsberg GE. Developmental and acclimatory contributions to water loss in a desert rodent: investigating the time course of adaptive change. Journal of Comparative Physiology B. 2001; 171: 669–679. 10.1007/s003600100218 11765976

[23] Schaefer J, Ryan A. Developmental plasticity in the thermal tolerance of zebrafish *Danio rerio*. Journal of Fish Biology. 2006; 69: 722–734. 10.1111/j.1095-8649.2006.01145.x

[24] West-Eberhard MJ. Developmental plasticity and evolution. Oxford University Press; 2003

[25] Nettle D, Bateson M. Adaptive developmental plasticity: what is it, how can we recognize it and when can it evolve? Proceedings of the Royal Society. B. 2015; 282: 20151005 10.1098/rspb.2015.1005 26203000 PMC4528519

[26] Huey RB, Berrigan D. Testing evolutionary hypotheses of acclimation. Animals and temperature: Phenotypic and evolutionary adaptation. 1996; 59: 205–237.

[27] Precht H. Concepts of the temperature adaptation of unchanging reaction systems of cold-blooded animals In: Prosser CL (Ed.), Physiological Adaptation. American Physiological Society, Washington, D.C 1958 pp. 50–78.

[28] Woods AH, Harrison JF. The beneficial hypothesis versus acclimation of specific traits: Physiological change in water-stressed Manduca sexta caterpillars. 2001; 74: 32–44. 10.1086/319302 11226012

[29] Woods AH, Harrison JF. Interpreting rejections of the beneficial acclimation hypothesis: when is physiological plasticity adaptive? Evolution. 2002; 56: 1963–1866 10.1111/j.0014-3820.2002.tb00201.x 12389732

[30] Leroi AM, Bennett AF, Lenski RE. Temperature acclimation and competitive fitness: An experimental test of the beneficial acclimation assumption. Proceedings of the National Academy of Sciences. 1994; 91: 1917–1921. 10.1073/pnas.91.5.1917 PMC43275 8127906

[31] Huey RB, Berrigan D, Gilchrist GW, Herron JC. Testing the adaptive significance of acclimation: A strong inference approach. American Zoology. 1999; 39: 323–336.

[32] Wilson RS, Franklin CE. Testing the beneficial acclimation hypothesis. Trends in Ecology & Evolution. 2002; 17: 66–70. 10.1016/S0169-5347(01)02384-9

[33] Caley MJ, Carr MH, Hixon MA, Hughes TP, Jones GP, Menge BA. Recruitment and the local dynamics of open marine populations. Annual Review of Ecology and Systematics. 1996; 27: 477–500.

[34] Moksnes PO. Interference competition for space in nursery habitats: density-dependent effects on growth and dispersal in juvenile shore crabs *Carcinus maenas*. Marine Ecology Progress Series. 2004; 281: 181–191. 10.3354/meps281181

[35] Newman RA. Ecological constraints on amphibian metamorphosis: interactions of temperature and larval density with responses to changing food level. Oecologia. 1998; 115: 9–16. 10.1007/s004420050485 28308472

[36] Andersen IL, Nævdal E, Bøe KE. Maternal investment, sibling competition, and offspring survival with increasing litter size and parity in pigs (*Sus scrofa*). Behavioral Ecology and Sociobiology. 2011; 65: 1159–1167. 10.1007/s00265-010-1128-4 21743767 PMC3096772

[37] Nilsson JÅ, Gårdmark A. Sibling competition affects individual growth strategies in marsh tit, *Parus palustris*, nestlings. Animal Behaviour 2001; 61: 357–365.

[38] Tewksbury JJ, Huey RB, Deutsch CA. Putting the heat on tropical animals. Science. 2008; 320: 1296 10.1126/science.1159328 18535231

[39] Hoffmann AA, Chown SL, Clusella-Trullas S. Upper thermal limits in terrestrial ectotherms: how constrained are they? Functional Ecology. 2013; 27: 934–949. 10.1111/j.1365-2435.2012.02036.x

[40] Gliman SE, Urban MC, Tewksbury J, Gilchrist GW, Holt RD. A framework for community interactions under climate change. Trends in Ecology and Evolution. 2010; 25: 325–331. 10.1016/j.tree.2010.03.002 20392517

[41] McCormick MI, Weaver CJ. It pays to be pushy: intracohort interference competition between two reef fishes. PLoS ONE. 2012; 7: e42590 10.1371/journal.pone.0042590 22900030 PMC3416846

[42] Grenchik MK, Donelson JM, Munday PL. Evidence for developmental thermal acclimation in the damselfish, *Pomacentrus moluccensis*. Coral Reefs. 2013; 32: 85–90. 10.1007/s00338-012-0949-1

[43] Allan BJM, Domenici P, McCormick MI, Watson S, Munday PL. Elevated CO2 affects predatory-prey interactions through altered performance. PLoS ONE. 2013; 8: e58520 10.1371/journal.pone.0058520 23484032 PMC3590170

[44] Allen GR. Damselfishes of the world. Mergus Publishers, Melle, Germany; 1991: P. 271

[45] McCormick MI, Moore JA, Munday PL. Influence of habitat degradation on fish replenishment. Coral Reefs 2010; 29: 537–546. 10.1007/s00338-010-0620-7

[46] McCormick MI. Lethal effects of habitat degradation on fishes through changing competitive advantage. Proceedings of the Royal Society B: Biological Sciences. 2012; 10.1098/rspb.2012.0854 22810432 PMC3427570

[47] Feary DA, Almany GR, McCormick MI, Jones GP. Habitat choice, recruitment and the response of coral reef fishes to coral degradation. Oecologia. 2007; 153: 727–737. 10.1007/s00442-007-0773-4 17566781

[48] McCormick MI, Meekan MG. Social facilitation of selective mortality. Ecology. 2007; 88: 1562–1570. 17601147 10.1890/06-0830

[49] Almany GR, Webster MS. The predation gauntlet: early post-settlement mortality in reef fishes. Coral Reefs. 2006, 25: 19–22. 10.1007/s00338-005-0044-y

[50] Australian Institute of Marine Science. Water temperature for January 2014, Arlington and Moore reefs. Long term Monitoring and Data Centre, AIMS. Viewed January 2014. http://data.aims.gov.au/aimsrtds/datatool.xhtml?from=2014-01-01&thru=2014-06-03&period=DAY&aggregations=MIN,MAX&channels=1751,100203,1896.

[51] Collins M, Knutti R, Arblaster J, Dufresne JL, Fichefet T, Friedlingstein P et al Long-term Climate Change: Projections, Commitments and Irreversibility. Climate Change 2013: The Physical Science Basis Contribution of Working Group I to the Fifth Assessment Report of the Intergovernmental Panel on Climate Change, [Stocker TF, Qin D, Plattner GK, Tignor M, Allen SK, Boschung J, Nauels A, Xia Y, Bex V, Midgley PM (eds.)]. Cambridge University Press, Cambridge, United Kingdom and New York, NY, USA; 2013.

[52] Gardiner NM, Munday PL, Nilsson GE. Counter-gradient variation in respirometry performance of coral reef fishes at elevated temperatures. PLoS ONE. 2010; 5: e13299 10.1371/journal.pone.0013299 20949020 PMC2952621

[53] Nilsson GE, Östlund-Nilsson S, Munday PL. Effects of elevated temperature on coral reef fishes: loss of hypoxia tolerance and inability to acclimate. Comparative Biochemistry and Physiology Part A: Molecular & Integrative Physiology. 2010; 156: 389–393. 10.1016/j.cbpa.2010.03.009 20233610

[54] Grenchik MK, Donelson JM, Munday PL. Evidence for developmental thermal acclimation in the damselfish, *Pomacentrus moluccensis*. Coral Reefs. 2013, 32: 85–90. 10.1007/s00338-012-0949-1

[55] Donelson JM, Munday PL, McCormick MI, Pitcher CR. Rapid, transgenerational acclimation of a tropical reef fish to climate change. Nature Climate Change. 2012; 2: 30–32. 10.1038/nclimate1323

[56] Poulos DE, McCormick MI. Who wins in the battle for space? The importance of priority, behavioural history and size. Animal Behaviour. 2014; 90: 305–314.

[57] Killen SS, Mitchell MD, Rummer JL, Chivers DP, Ferrari MC, Meekan MG et al Aerobic scope predicts dominance during early life in a tropical damselfish. Functional Ecology. 2014; 28: 1367–1376.

[58] McCormick MI. Behaviourally Mediated Phenotypic Selection in a Disturbed Coral Reef Environment. PLoS ONE. 2009; 4: e7096 10.1371/journal.pone.0007096 19763262 PMC2740825

[59] Williams SE, Shoo LP, Isaac JL, Hoffmann AA, Langham G. Towards an integrated framework for assessing the vulnerability of species to climate change. PLoS Biology. 2008; 6: e325 10.1371/journal.pbio.0060325 19108608 PMC2605927

[60] Kordas RL, Harley CD, O'Connor MI. Community ecology in a warming world: the influence of temperature on interspecific interactions in marine systems. Journal of Experimental Marine Biology and Ecology. 2011; 400: 218–226.

[61] Taniguchi Y, Rahel FJ, Novinger DC, Gerow KG. Temperature mediation of competitive interactions among three fish species that replace each other along longitudinal stream gradients. Canadian Journal of Fisheries and Aquatic Sciences. 1998; 55: 1894–1901. 10.1139/f98-072

[62] Hoffman AA. Acclimation: increasing survival at a cost. Trends in Ecology & Evolution. 1995; 10:1–2. 10.1016/s0169-5347(00)88949-1

[63] Levins R. Thermal acclimation and heat resistance in Drosophila species. The American Naturalist. 1969; 103: 483–499.

[64] DeWitt TJ, Sih A, Wilson DS. Costs and limits of phenotypic plasticity. Trends in Ecology & Evolution. 1998; 13: 77–81. 10.1016/s0169-5347(97)01274-3 21238209

