# Supplementary material for: Duration of Exposure to Elevated Temperature Affects Competitive Interactions in Juvenile Reef Fishes
Source: PLoS One. 2016 Oct 13;11(10):e0164505. doi: 10.1371/journal.pone.0164505 (PMC5063334; doi:10.1371/journal.pone.0164505)
Supplement: S1 Fig — Test temperature listed horizontally and split by 4d (grey) and 90d exposure treatments (open). Intraspecific contests of Pomacentrus amboinensis (a) with after 4d exposure to elevated temperature had no change in winner aggression score compared to controls, but 90d exposure had significantly lower winner scores when compared to 4d (F1,38 = 7.79, P = 0.008). Aggression scores for Pomacentrus moluccensis (b) increased from control after 4d exposure to elevated temperature (F2,27 = 3.46, P = 0.046), but there was no difference between 4d and 90d. Aggression in interspecific contests (c) increased slightly with temperature after 4d, but was reduced to control levels after 90d exposure (F1,38 = 5.14, P = 0.029). Significance of p < 0.05 symbolised with (*). (DOCX) [file pone.0164505.s002.docx]

*

*

*

*


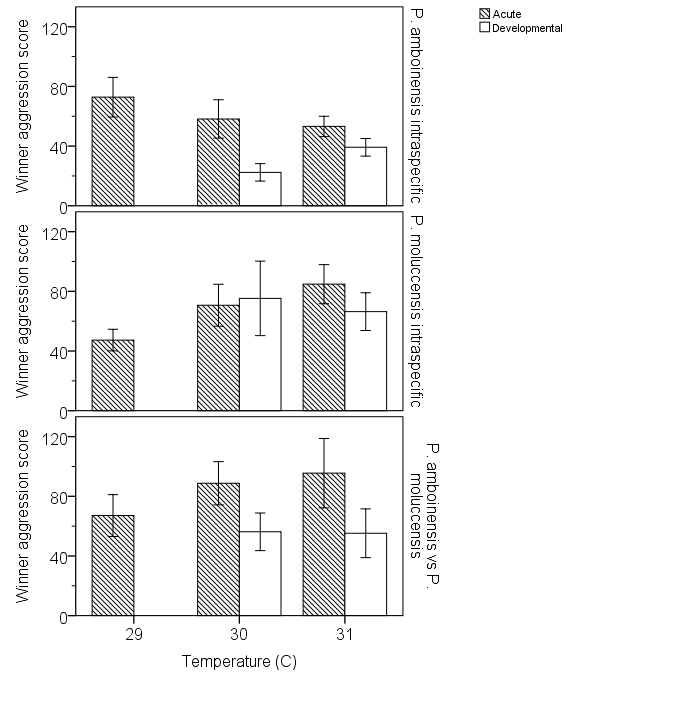


*a*

*b*

*c*

**S1 Fig.** **Aggression scores ± SE of contest winner.** Test temperature listed horizontally and split by 4d (grey) and 90d exposure treatments (open). Intraspecific contests of *Pomacentrus amboinensis* (*a*) with after 4d exposure to elevated temperature had no change in winner aggression score compared to controls, but 90d exposure had significantly lower winner scores when compared to 4d (F_1,38_ = 7.79, P = 0.008). Aggression scores for *Pomacentrus moluccensis* (*b*) increased from control after 4d exposure to elevated temperature (F_2,27_ = 3.46, P = 0.046), but there was no difference between 4d and 90d. Aggression in interspecific contests (*c*) increased slightly with temperature after 4d, but was reduced to control levels after 90d exposure (F_1,38_ = 5.14, P = 0.029). Significance of p < 0.05 symbolised with (*).
