# Supplementary material for: Duration of Exposure to Elevated Temperature Affects Competitive Interactions in Juvenile Reef Fishes
Source: PLoS One. 2016 Oct 13;11(10):e0164505. doi: 10.1371/journal.pone.0164505 (PMC5063334; doi:10.1371/journal.pone.0164505)
Supplement: S2 Fig — Calculated as sum of attacks, displays, and avoids for both competitors. Test temperature listed horizontally, and split by 4d (grey) and 90d exposure treatments (open). Intraspecific contests of Pomacentrus amboinensis (a) had a non-significant reduction of interactions after 4d exposure to temperature compared to controls but 90d exposed treatments had significantly less interactions than 4d (F1,38 = 4.17, P = 0.048). Contests with Pomacentrus moluccensis (b) had more interactions with elevated temperature after 4d, but showed no difference after 90d. Interspecific treatments (c) had increased interactions with temperature after 4d, but 90d exposure reduced this back to control levels (F1,38 = 6.3, P = 0.016). Significance P < 0.05 symbolised with (*). (DOCX) [file pone.0164505.s003.docx]

*

*

*

*

*c*

*b*

*a*


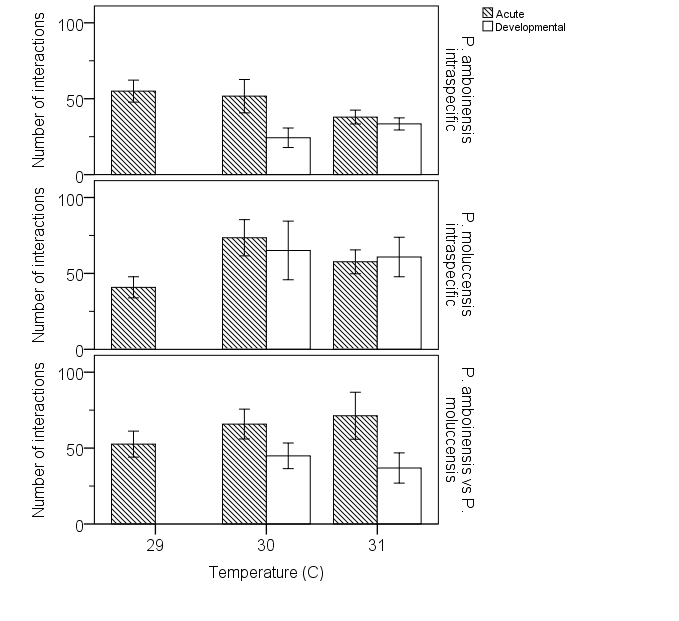


**S2 Fig. Total number of interactions ± SE by both competitors per contest.** Calculated as sum of attacks, displays, and avoids for both competitors. Test temperature listed horizontally, and split by 4d (grey) and 90d exposure treatments (open). Intraspecific contests of *Pomacentrus amboinensis* (*a*) had a non-significant reduction of interactions after 4d exposure to temperature compared to controls but 90d exposed treatments had significantly less interactions than 4d (F_1,38_ = 4.17, P = 0.048). Contests with *Pomacentrus moluccensis* (*b*) had more interactions with elevated temperature after 4d, but showed no difference after 90d. Interspecific treatments (*c*) had increased interactions with temperature after 4d, but 90d exposure reduced this back to control levels (F_1,38_ = 6.3, P = 0.016). Significance P < 0.05 symbolised with (*).
