# Supplementary material for: Duration of Exposure to Elevated Temperature Affects Competitive Interactions in Juvenile Reef Fishes
Source: PLoS One. 2016 Oct 13;11(10):e0164505. doi: 10.1371/journal.pone.0164505 (PMC5063334; doi:10.1371/journal.pone.0164505)

**S1 Table.** Experimental design matrix. Aim 1 compared control (grey) fish to individuals with 4d exposure (solid) to elevated temperatures. Aim 2 compared contests using 4d exposed fish with 90d exposure treatments (open).


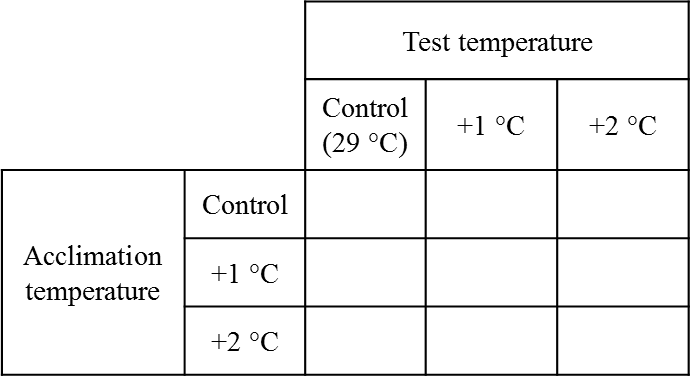

Supplement: S1 Table — Aim 1 compared control (grey) fish to individuals with 4d exposure (solid) to elevated temperatures. Aim 2 compared contests using 4d exposed fish with 90d exposure treatments (open). (DOCX) [file pone.0164505.s004.docx]
